# Differential effects of remotely supervised transcranial direct current stimulation on recognition memory depending on task order

**DOI:** 10.3389/fnhum.2023.1239126

**Published:** 2023-08-09

**Authors:** Casey M. Imperio, Elizabeth F. Chua

**Affiliations:** ^1^Department of Psychology, Brooklyn College, Brooklyn, NY, United States; ^2^Department of Psychology, The Graduate Center of the City University of New York, New York, NY, United States

**Keywords:** remotely supervised tDCS, DLPFC, metamemory, episodic memory, semantic memory

## Abstract

**Background:**

Prior work has shown positive effects of High Definition transcranial direct current stimulation (HD-tDCS) over the dorsolateral prefrontal cortex (DLPFC) on semantic memory performance and metamemory monitoring accuracy. However, HD-tDCS requires setup by a trained researcher, which is not always feasible. Few studies have used remotely supervised (rs) tDCS in healthy populations, and remote supervision has strong practical benefits.

**Objective/hypothesis:**

The goal of the current study was to test if previously shown effects of HD-tDCS over the left DLPFC on semantic memory performance and metamemory monitoring accuracy extended to conventional rs-tDCS, which is less focal than HD-tDCS, and to episodic memory and metamemory tasks.

**Materials and methods:**

A total of 36 healthy participants completed 6 weeks of rs-tDCS sessions, with either active left or right anodal DLPFC stimulation, or sham. Participants completed semantic and episodic memory and metamemory tasks, which each lasted for three consecutive sessions, and session order was counterbalanced across participants.

**Results:**

Overall, there were no main effects of rs-tDCS on metamemory monitoring accuracy or memory performance for either the semantic or the episodic tasks. However, there were effects of rs-tDCS that depended on the order of completing the episodic and semantic task sessions. When participants completed the semantic task sessions after the episodic task sessions, semantic recognition was greater in the left anodal DLPFC condition. In a parallel effect, when participants completed the episodic task sessions after the semantic task sessions, episodic recognition was greater in the right anodal DLPFC condition.

**Conclusion:**

Prior experience with tDCS is a factor for effects of rs-tDCS on cognition. Additionally, the current experiment provides evidence for the feasibility of fully remotely supervised tDCS in healthy participants.

## Introduction

Remembering general facts (i.e., semantic memory) and personal events (i.e., episodic memory) are both critical for daily functioning, and so is the ability to monitor our own memory. Assessing the contents of memory is part of what is referred to as metamemory monitoring, and our subjective assessments of memory are often diagnostic of objective memory performance (Nelson and Narens, 1990). One common metamemory task involves making feeling-of-knowing (FOK) ratings, which are judgments given about the future memorability of some sought-after piece of information that is currently unrecallable (Hart, 1965). Brain imaging studies have shown that activity in the left and right dorsolateral prefrontal cortex (DLPFC) correlates with memory retrieval (Cabeza and Nyberg, 2000; Tulving, 2002; Maril et al., 2005; Blumenfeld and Ranganath, 2007; Ubaldi et al., 2022) and with FOK ratings for episodic and semantic tasks (Kikyo et al., 2002; Luo et al., 2004; Reggev et al., 2011). Furthermore, studies have shown that applying high definition transcranial direct current stimulation (HD-tDCS) over the left DLPFC during a semantic memory and metamemory task leads to increased semantic retrieval (Chua et al., 2017), as well as increased metamemory monitoring accuracy (Chua and Ahmed, 2016; Chua et al., 2017). To extend prior work, this experiment tested if anodal conventional tDCS over the left and right DLPFC improved both semantic and episodic memory retrieval and metamemory monitoring accuracy. Data collection started during the COVID-19 pandemic, when in-person research was not allowed, so remotely supervised tDCS (rs-tDCS), in which tDCS was supervised over video chat, was used. rs-tDCS has the potential to be an incredibly useful tool in both research and clinical interventions (Charvet et al., 2020; Gough et al., 2020; Shaw et al., 2020; Pilloni et al., 2022; Richardson et al., 2023); the device has been used from the comfort of one’s own home, after an initial in-office training session (Shaw et al., 2020). Ancillary goals were to test: (1) the feasibility of fully at home rs-tDCS, with the initial training session being held via video conferencing, in healthy participants, (2) the feasibility of participants completing multiple experiments with rs-tDCS, and (3) to examine potential order effects in multiple experiment designs with tDCS.

### Types of tDCS

Prior work showed that HD-tDCS over the left DLPFC increased semantic retrieval (Chua et al., 2017) and increased metamemory monitoring accuracy (Chua and Ahmed, 2016; Chua et al., 2017), and this experiment tests whether this extends to conventional tDCS. Although HD-tDCS is more focal than conventional tDCS, in relation to modifying cognition, results have been mixed for both conventional tDCS (Berlim et al., 2013; Galli et al., 2019; Inagawa et al., 2019) and HD-tDCS (Müller et al., 2022). Some studies comparing conventional to HD-tDCS have shown that HD-tDCS results in better cognitive outcomes (Gözenman and Berryhill, 2016), while others have shown no differences in cognition based on device type (Hogeveen et al., 2016). It is still an open question whether and when the same effects will be shown with conventional and HD-tDCS. Therefore, the current experiment aims to determine if findings from HD-tDCS studies on memory and metamemory (Chua and Ahmed, 2016; Chua et al., 2017) can be extended to conventional tDCS.

A practical drawback for widespread use of both conventional and HD-tDCS as a clinical intervention is that it requires a trained individual to properly setup the electrodes and monitor stimulation. This means that participants must come in to the office for their sessions (Cucca et al., 2019), and this may be especially difficult for patient populations (Charvet et al., 2015). rs-tDCS was developed so that brain stimulation could be delivered from the comfort of one’s own home (Charvet et al., 2020; Shaw et al., 2020; Pilloni et al., 2022; Richardson et al., 2023), which could reduce the attrition issues in multisession studies with both patient and healthy populations. rs-tDCS, which has been shown as safe, tolerable, and feasible in different patient populations (Dobbs et al., 2018; Cucca et al., 2019; Charvet et al., 2020; Mishra and Thrasher, 2021; Cappon et al., 2022; Pilloni et al., 2022; Richardson et al., 2023), typically involves an initial in-office training session (Charvet et al., 2020; Gough et al., 2020; Pilloni et al., 2022), which in some circumstances, may be necessary. However, one study did show that virtual training for rs-tDCS administration was possible in adults with major depressive disorder (Cappon et al., 2022). Another study used either in-office or remote training sessions for the initial visit (Pilloni et al., 2022). Thus, a secondary goal of this study was to assess the feasibility of a remote training session for the tDCS setup and administration in healthy participants. This was especially important when the current experiment took place because in-office research was not feasible due to the COVID-19 pandemic.

### The current study

The current experiment tested the effects of anodal rs-tDCS over the left and right DLPFC in episodic and semantic memory and metamemory. The goal was to extend prior work showing that HD-tDCS over the left DLPFC resulted in improved metamemory monitoring accuracy (Chua and Ahmed, 2016; Chua et al., 2017) and semantic retrieval (Chua et al., 2017), to remotely supervised, conventional tDCS, and to an episodic task. For the semantic task, we expected that the left anodal DLPFC stimulation would result in improvements in semantic retrieval (Chua et al., 2017) and metamemory monitoring accuracy (Chua and Ahmed, 2016; Chua et al., 2017), as shown in prior HD-tDCS work. Turning to the episodic task, we expected that right anodal DLPFC stimulation would lead to improved episodic retrieval (Tulving, 2002; Blumenfeld and Ranganath, 2007) and metamemory monitoring accuracy (Reggev et al., 2011).

Although many within subjects tDCS studies have not shown order effects (Fregni et al., 2005; Pereira et al., 2013; Barbieri et al., 2016), many multi-session tDCS studies have shown effects of time (André et al., 2016; Perceval et al., 2020; Au et al., 2022); therefore, because two experimental tasks with multiple stimulation sessions were used, we also examined potential order effects. This is important because most studies have not used multiple behavioral tasks with different stimulation sessions.

## Materials and methods

### Participants

This experiment and the procedures described below were approved by the Human Research Protection Program at the City University of New York (CUNY). Participants were recruited via online postings on a clinicaltrials.gov, virtual class announcements, and Facebook posts. Eligibility criteria required participants to be right hand dominant and between the ages of 18 and 35, with normal or corrected to normal vision and hearing, having learned English before the age of five, with no: chronic skin conditions or unhealed wounds on the forehead or scalp, history of mental illness, learning disability, heart disease, seizures or epilepsy, neurological or movement disorders, drug or alcohol abuse, prescription medications that cause CNS changes, prescription anti-depressant medications. Female participants could not be pregnant or lactating. Forty-nine participants gave written informed consent before the first experimental session, were informed that their participation was voluntary, and that they could withdraw at any time without loss of benefits. Nine participants withdrew before receiving the device, one could not complete the rs-tDCS training, one could not tolerate tDCS, one felt uncomfortable continuing, and one no longer had time. The final sample was 36 participants ages, 18–31 (*M* = 23.69, *SD* = 3.68). A power analysis using G*Power determined that 36 participants were obtained an effect size (f) of 0.21, with an alpha of 0.05 and 80% power (Faul et al., 2009).

### Behavioral tasks

**FIGURE 1:**
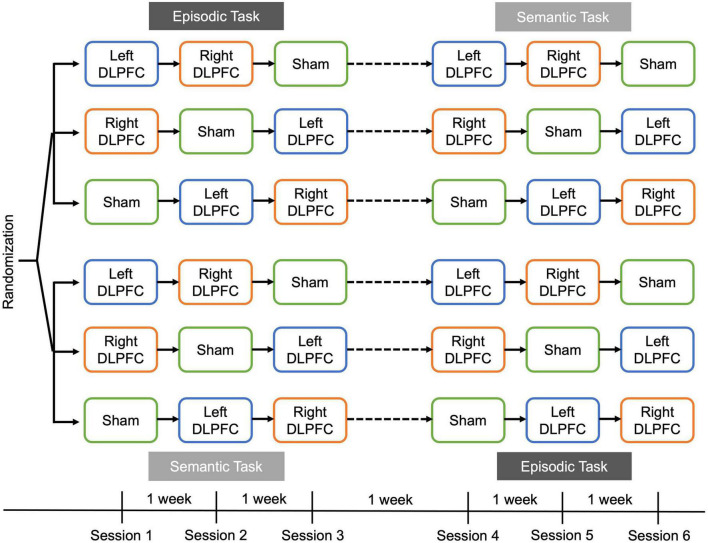
Overall task design. Participants were randomized to complete either the episodic task or semantic task first, in a cross-over design. Sessions were 1 week apart, and participants completed a recall-judgment-recognition memory task in each session (Figure 2). During a session, participants received either **left** anodal DLPFC (blue), **right** anodal DLPFC (orange), or sham stimulation (green). The order of stimulation condition was counterbalanced across participants and was consistent within a participant such that the same order was received for the episodic and semantic tasks.

After determining participants were eligible, and consenting via video conference, the materials for administering rs-tDCS were shipped to the participant. Our original research goal was to compare the effects of left anodal DLPFC, right anodal DLPFC and sham conventional tDCS on episodic metamemory and retrieval, and then on semantic metamemory and retrieval. However, because we pivoted to rs-tDCS because in person data collection was not allowed due to COVID-19, participants completed both the episodic and semantic tasks in a cross-over design (Figure 1). Thus, the study included two separate within-subjects experiments (one semantic and one episodic) that each had one session of left anodal DLPFC, right anodal DLPFC, and sham, for a total of six sessions. Each session was at least 1 week apart, and scheduled at approximately the same time of day. The task order, as well as test versions for each task (A,B, or C), were randomly assigned, and each task occurred over three consecutive weeks.

#### Episodic task

**FIGURE 2:**
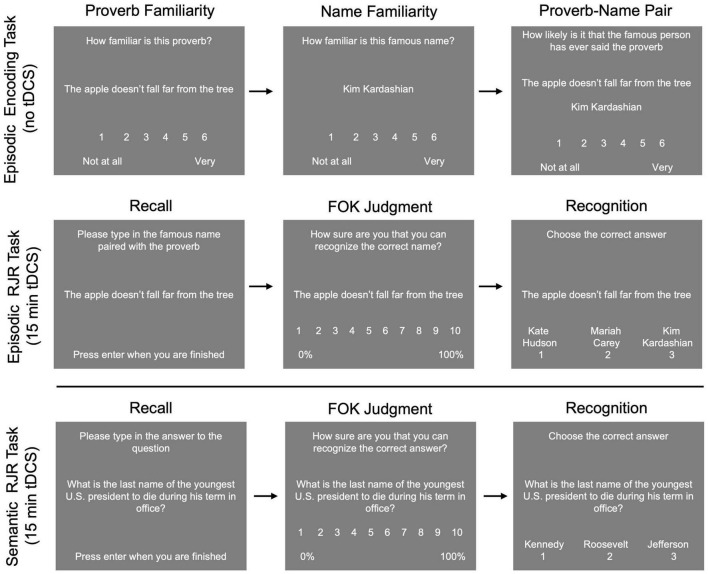
Example stimuli and trials for the episodic **(top)** and semantic tasks **(bottom)**. Participants completed a proverb-name associative encoding task in which they rated familiarity with the proverb, then familiarity with the name, and then the likelihood that the person had ever said the proverb. After encoding, tDCS was applied during the episodic recall-judgment-recognition task. For the semantic task, tDCS was applied during the recall-judgment-recognition task.

Each version contained a unique set of 100 proverb/famous name pairs. Proverbs and famous names were chosen and assigned to lists matched on familiarity ratings from pilot data. The episodic task consisted of two blocks: (1) Encoding and (2) Recall-Judgment-Recognition (RJR; Figure 2, top). During the encoding block, participants rated the familiarity of the proverb using a scale of 1 (not at all familiar) to 6 (very familiar). Next, they rated the famous name using the same scale. Finally, they rated how likely it was that the famous person had ever said the proverb, using a scale of 1 (Not at all likely) to 6 (Very likely). Each proverb, name, and pairing were shown for 3 s, with a 1 s fixation cross between each presentation. Participants were instructed to read the stimuli silently and attempt to remember the pairing. The encoding phase lasted ∼25 min.

After encoding, participants were given the RJR block with all 100 pairs, and left anodal DLPFC, right anodal DLPFC, or sham rs-tDCS was applied for 15 min (see rs-tDCS methods for details). For this task, we were interested in the effects of stimulation on metamemory and retrieval, thus, stimulation was applied after encoding, during the RJR task. During the RJR task, participants were shown the proverb and asked to recall and type in the associated name. If they could not recall the name, they were instructed to type in “idk” for “I don’t know.” Then, participants gave an FOK rating, in which they indicated how likely they thought they were to recognize the name previously paired with the proverb later, on a 1 (0–10% sure in ability to recognize the correct answer) to 10 scale (91–100% sure in ability to recognize the correct answer), with the other numbers representing increments of increasing certainty. Finally, participants completed a three item (one correct, two incorrect) forced choice recognition test for the proverb/name pair, in which they were asked to select the previously paired name or guess if they were unsure.

#### Semantic task

In each semantic task session, participants were presented with a set of 100 unique general knowledge questions from the Baruch Knowledge Norms (BK-Norms)^1^ one-at-a-time on a computer screen. The BK-Norms is a database of 406 general knowledge questions spanning subjects such as math, science, arts, geography, and history. Each question was paired with three answer choices: the correct answer and the two most commonly given different incorrect answers from the BK-norms database.

For the semantic task, participants were given instructions, and then completed the RJR task (Figure 2, bottom), during which stimulation was applied. First, participants were shown a general knowledge question, and asked to recall the correct answer to the question. If they could not recall the correct answer, they were instructed to type in “idk” for “I don’t know.” Then, participants gave an FOK rating, using the same scale as in the episodic task. Finally, participants completed a three item (one correct, two incorrect) forced choice recognition test for the general knowledge question where they were asked to select the correct answer, and if they did not know they were to guess.

### rs-tDCS methods

In a double-blind design, participants were randomly assigned to a condition schedule with two active and one sham rs-tDCS condition per task (Figure 1). The rs-tDCS montages included active left or right anodal DLPFC stimulation, and sham rs-tDCS, with half of the participants receiving sham rs-tDCS over the left DLPFC, and half receiving sham rs-tDCS over the right DLPFC. Stimulation order and test version order were counterbalanced and were the same for both the episodic and semantics tasks for each participant.

Remotely supervised transcranial direct current stimulation was administered using the Soterix 1 × 1 tDCS mini-CT (Model 1601-LTE, Soterix Medical, New York, NY, USA). The montage was set up via the Soterix omni-lateral-electrode (OLE) SNAPstrap, which, through forward modeling studies, was optimized for the DLPFC stimulation and has been shown to be more accurate in constraining stimulation to the DLPFC compared to traditional DLPFC montages (Seibt et al., 2015). This head strap does not align to F3 and F4 like other DLPFC montages, and the SNAPstrap has fixed electrode positions and strap angles to ensure accurate placement of the electrodes when the occipital strap is centered over the inion. Participants were instructed on how to measure their head, and on how to find the inion, to ensure the correct size SNAPstrap and accurate placement. For each session, this was done while on video conference with a researcher to increase accuracy. The anode and cathode electrodes were inserted into 5 cm × 5 cm saline-soaked SNAPpads (Soterix Medical). Because the mini-CT was designed with remote supervision in mind, there are numerous safety and control features. The device will only apply current when the participant enters a single use code, so participants were not able to use the device other than during the sessions. The researcher gave the participant the code for the session over video conference.

For active stimulation, the current ramped up to 2 mA over a 30 s interval, then stayed at this current for the duration of the stimulation (15 min), ramping back down to 0 mA during the last 30 s. For sham tDCS, the current ramped up to 2 mA over a 30 s interval, then ramped back down to 0 mA for the duration of the stimulation, and repeated the ramp up and down process during the last 30 s. About halfway through the session, as well as at the end of stimulation, the researcher asked the participant to report contact quality of the electrodes.

Participants were trained during their first experimental session, by the researcher, via video conferencing (∼30 min). Participants viewed pre-recorded videos about: (1) the rs-TDCS materials, and (2) how to set up the device and screenshots of the device. Then, participants asked questions, and set up the head strap and device on themselves. The researcher supervised, giving instructions when needed, and answering questions. Throughout the training session, participants were instructed to monitor their contact quality, which could be categorized as “good,” “moderate,” or “poor.” The device is programmed such that stimulation can only be activated when the electrodes are at the most optimal contact quality (i.e., “good”). When the head strap was placed correctly, participants received a short amount of tDCS to determine if they could tolerate stimulation, in which the current ramped up to 2 mA. Typically, when using the Soterix conventional 1 × 1 tDCS device, a pre-stim tickle, which consists of applying 1 mA of stimulation for 30 s, is administered to assess if participants can tolerate stimulation (Soterix Medical Inc, 2015). However, the 1 × 1 tDCS mini-CT device does not include a pre-stim tickle option, so to assess participant tolerability of stimulation, we programmed pre-stim tickle sessions using the available stimulation parameters. For the pre-stim tickle, the device was set to ramp up 2 mA of current for 5 min, which was the shortest stimulation duration that can be programmed with the 1 × 1 mini-CT device. After about ∼ 1–2 min, the researcher asked the participant if the stimulation was tolerable and aborted the stimulation, ending the training session. Participants were given another chance to ask questions, and then the experimental session started. To ensure contact quality remained consistent throughout the experiment, the researcher asked the participant to report the contact quality both halfway through the stimulation (∼ 7.5 min), and at the end of the stimulation (15 min).

At the end of each experimental session, participants filled out an online questionnaire, and were asked if they experienced any of the following side effects of rs-tDCS: headache, neck pain, scalp pain, tingling, burning, skin redness, sleepiness, trouble concentrating and acute mood change [adapted from Charvet et al. (2015) and Shaw et al. (2020)]. They were asked to rate the severity of the side effects on a scale of 1 (absent) to 10 (severe), as well as rate their perception of the relationship between each sensation and stimulation on a scale of 1 (none) to 5 (definitely). Finally, participants were asked to indicate if they believed that they received active or sham stimulation.

### Data analysis

Before the data were analyzed, two researchers checked free recall and cued recall responses for correctness. Any discrepancies were resolved by researcher 1. Interrater reliability was assessed using Cohen’s kappa and showed that for both free recall on the semantic task, and cued recall on the episodic task, researchers agreed 99% of the time. To determine if individual reactions to rs-tDCS influenced the results, tolerability, sensations, and blinding of stimulation was assessed between stimulation conditions. Friedman tests were used to assess differences in tolerability and sensations, and Wald Chi-square tests were used to determine if blinding was adequate. Only sensations that were attributed to rs-tDCS were analyzed (e.g., headache that was not indicated as being related to rs-tDCS was not analyzed). For the sham condition, half of the participants received left anodal DLPFC stimulation, and the other half right anodal DLPFC stimulation. Analyses were conducted to determine if there were any differences in recall accuracy, recognition accuracy, and FOK ratings between sham montages. No differences were found between sham montages for both the semantic and episodic tasks (all *p*’s > 0.10), and thus, all analyses were conducted with sham collapsed across montages.

We also tested whether memory performance was matched across test version and session order using repeated measures ANOVAs on free recall, cued recall, and recognition. For the semantic task, there was a significant difference in recall accuracy by test version [*F*_(2, 68)_ = 3.904, *p* = 0.025], but not recognition (unrecalled trials only), [*F*_(2, 68)_ = 1.915, *p* = 0.155]. For the episodic task, there were no differences in recall or recognition accuracy by test version (all *p*’s > 0.30).

Due to the inclusion of two separate within subjects experiments, analyses on both memory and metamemory performance were divided by task type (semantic vs. episodic). For both tasks, to assess the effect of HD-tDCS on memory and metamemory performance, several mixed ANOVAs on recall and recognition accuracy, as well as average FOK ratings and metamemory monitoring accuracy were conducted. FOK ratings are defined as a sense that a currently unrecallable item is in memory (Hart, 1965), and thus, for analyses concerning both FOK ratings and metamemory monitoring accuracy, only trials where the participant was incorrect at the initial recall were used. Metamemory monitoring accuracy was determined by computing a measure based in signal detection theory, d_*a*_, which compares metacognitive hits to misses (Benjamin and Diaz, 2008). Mixed ANOVAs were conducted to assess metamemory monitoring accuracy. One participant was removed from semantic task analysis, and two from episodic task analysis because we could not calculate d_*a*_ due to low trial counts. For all analyses, task order, which refers to whether each task was received 1st or 2nd (i.e., semantic task 1st vs. semantic task 2nd and episodic task 1st vs. episodic task 2nd), was included to determine if the order in which the tasks were completed had any effect on our outcome measures. If any ANOVAs violated sphericity, the Greenhouse Geisser correction was reported.

## Results

### Feasibility of remotely supervised training

Feasibility was assessed based on the number of participants who were able to successfully setup tDCS and maintain good contact quality throughout stimulation. The majority of participants (39 out of 40) were able to adequately follow the training videos and instructions that were given remotely during the tDCS training session. These 39 participants passed all quality checks for self-administering tDCS using the mini-CT device and were able to properly adhere the sponges to the headstrap, position the headstrap, enter the researcher provided code to begin stimulation, and monitor contact quality. One participant was removed from the study due to the inability to follow instructions during the training session. During the sessions, all participants who passed the training were able to achieve and maintain good contact quality throughout their sessions. There were no reports of contact quality readings other than good. For higher resolution information about quality, the mini-CT also records events where contact quality was poor or the device paused, and 209/216 sessions had 0 events. Of the seven sessions that had events, there was 1 pause for 4 min 56 s, and 13 events where quality dropped to poor, with a range of 1–48 s of poor quality in the 7 sessions (*M* = 19.7, *SD* = 17.6). Thus, we conclude that at least for our sample, fully remotely supervised tDCS, without an in-person training session, may be feasible in healthy younger adults.

Although not a unique issue to remote supervision, attrition and schedule adherence is also an issue we examined. A total of 36 participants completed all 6 sessions (see Table 1 for full demographic information). Participants were able to choose the time of their sessions, which could take place during the morning (6:00 a.m.–11:59 a.m. EST), afternoon (12:00 p.m.–5:59 p.m. EST), or evening (6:00 p.m.–11:59 p.m. EST). Ten participants had morning sessions, 11 had afternoon sessions, and 15 had evening sessions. Out of the 36 participants, 16 were able to complete their six sessions at the exact same time of day (3 morning, 5 afternoon, and 8 evening). On average, start time between the six sessions deviated by 34 min.

**TABLE 1:** Demographic information for final sample.

	Ethnic categories									
	**Not Hispanic or Latino**			**Hispanic or Latino**			**No reported ethnicity**			
**Racial categories**	**Female**	**Male**	**Unknown**	**Female**	**Male**	**Unknown**	**Female**	**Male**	**Unknown**	**Total**
Asian	13	4	0	0	0	0	1	0	0	18
Black/African American	3	0	0	1	0	0	0	0	0	4
White/Caucasian	3	5	1	2	1	0	0	0	0	12
Multiracial	0	1	0	0	0	0	0	0	0	1
Prefer not to answer	1	0	0	0	0	0	0	0	0	1
Total	20	10	1	3	1	0	1	0	0	36

### Stimulation sensations and subject blinding

Most participants tolerated rs-tDCS well and only experienced mild to moderate side effects (Table 2), with occasional “severe” side effects (Table 3). Friedman’s tests were conducted on sensations that were attributed to rs-tDCS (e.g., headache that was not indicated as being related to rs-tDCS was not counted) and showed for the semantic task, there was a significant difference in tingling sensations reported (χ^2^ = 6.16, *p* = 0.046), with more people reporting tingling sensations for both left anodal DLPFC and right anodal DLPFC compared to sham (all *p*’s < 0.05). There were no other differences in reported sensations by stimulation conditions for the semantic task (all *p*’s > 0.05). For the episodic task, there was a significant difference in burning sensations by stimulation condition (χ^2^ = 7.55, *p* = 0.023), with greater reports of burning sensations for left anodal DLPFC compared to sham (*p* = 0.007), and marginally greater reports of burning sensations for right anodal DLPFC compared to sham (*p* = 0.058). For the episodic task, there were no other significant differences in sensations by stimulation condition (all *p*’s > 0.05).

**TABLE 2:** Reports of mild to moderate side effects attributed to stimulation.

	Episodic			Semantic		
	**Left anodal DLPFC**	**Right anodal DLPFC**	**Sham**	**Left anodal DLPFC**	**Right anodal DLPFC**	**Sham**
Headache	13	9	8	5	9	6
Neck pain	2	1	2	0	0	1
Scalp pain	7	10	10	6	5	5
Itching	20	18	19	21	17	13
Tingling	23	24	21	27	22	23
Burning	17	22	13	17	21	17
Skin redness	8	11	12	5	8	8
Sleepiness	10	5	6	5	6	3
Trouble concentrating	12	11	14	8	10	8
Mood changes	0	1	3	2	3	1
Other	3	3	3	0	1	2

**TABLE 3:** Reports of severe side effects attributed to stimulation.

	Episodic			Semantic		
	**Left anodal DLPFC**	**Right anodal DLPFC**	**Sham**	**Left anodal DLPFC**	**Right anodal DLPFC**	**Sham**
Headache	0	2	2	0	0	0
Neck pain	0	0	0	0	0	0
Scalp pain	2	1	2	0	0	0
Itching	2	4	3	0	3	2
Tingling	5	7	5	5	5	2
Burning	6	1	3	2	3	0
Skin redness	0	0	0	0	1	0
Sleepiness	3	2	2	0	0	1
Trouble concentrating	3	3	0	0	1	0
Mood changes	1	1	0	0	0	0
Other	0	0	0	0	0	0

At the end of each session, participants indicated whether they thought the received active or sham stimulation. Wald Chi-square tests (see Table 4) showed a significant difference in blinding between *right anodal DLPFC* and sham (Wald Chi-square = 6.456, *p* = 0.011), with 31 participants guessing active, and 5 guessing sham during *right anodal DLPFC* stimulation, and 22 participants guessing active and 14 guessing sham during sham. This indicates that participants were better able to identify the *right anodal DLPFC* condition as active compared to sham. No other conditions differed in terms of subject blinding.

**TABLE 4:** Participant blinding by task.

	Comparison	Wald chi-square	*p*-values
Semantic task	*Left anodal* vs. *Right anodal DLPFC*	0.200	0.655
	*Left anodal DLPFC* vs. Sham	2.627	0.105
	*Right anodal DLPFC* vs. Sham	1.977	0.160
Episodic task	*Left anodal* vs. *Right anodal DLPFC*	0.332	0.565
	*Left anodal DLPFC* vs. Sham	2.899	0.089
	*Right anodal DLPFC* vs. Sham	6.456	0.011*

*Denotes significance at *p* < 0.05.

### The effects of rs-tDCS on memory

**FIGURE 3:**
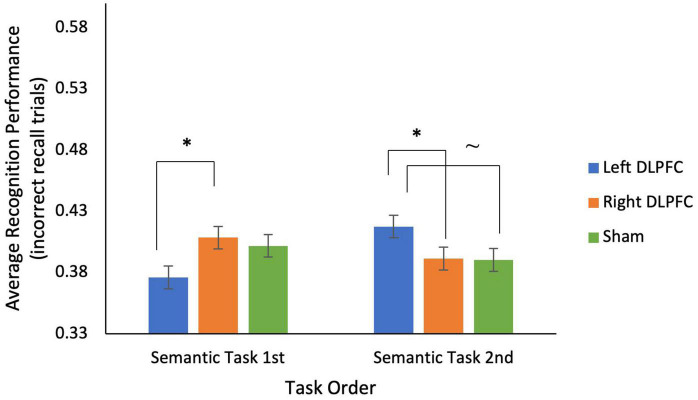
There was a significant interaction between stimulation condition and task order for recognition performance on trials incorrectly recalled for the semantic task. For participants who completed the semantic task 1st, during sessions 1–3, recognition performance was significantly lower for *left* *anodal DLPFC* stimulation compared to *right* *anodal DLPFC* stimulation. For participants who completed the semantic task 2nd, during sessions 4–6, recognition performance was significantly higher for *left* *anodal DLPFC* stimulation compared to *right* *anodal DLPFC* stimulation, and marginally higher than sham. *p < 0.05, ∼ indicates marginal significance (*p* = 0.065).

Several mixed ANOVAs, with stimulation condition as a within-subjects factor, and task order (i.e., semantic task 1st vs. 2nd, episodic task 1st vs. 2nd) as a between subjects factor, were conducted to examine memory and metamemory (see Table 5). For the semantic task, 2 (semantic task 1st vs. 2nd) × 3 (*Left anodal DLPFC* vs. *Right anodal DLPFC* vs. sham) mixed ANOVAs showed no main effect of stimulation condition, and no main effect of test order on either recall or recognition (for non-recalled trials) (all *p*’s > 0.30). There was no interaction between task order and stimulation condition on recall, however, there was a significant interaction between stimulation condition and task order on recognition [*F*_(2, 66)_ = 3.405, *p* = 0.039; see Figure 3]. This interaction was driven by better recognition performance when the semantic task was 2nd (*M* = 0.42, *SD* = 0.07) compared to when the semantic task was 1st (*M* = 0.38, *SD* = 0.06) in the *left anodal* DLPFC condition: *t*(33) = −1.839, *p* = 0.037, but not for *right DLPFC* and sham (all *p*’s > 0.05). Additionally, participants who received the semantic task 1st had significantly greater recognition performance for *right anodal DLPFC* (*M* = 0.41, *SD* = 0.04) compared to *left anodal DLPFC* (*M* = 0.38, *SD* = 0.06) stimulation [*t*(17) = −1.939, *p* = 0.035], whereas participants who received the semantic task 2nd had significantly greater recognition performance for *left anodal DLPFC* (*M* = 0.42, *SD* = 0.07) compared to *right anodal DLPFC* (*M* = 0.39, *SD* = 0.06) stimulation [*t*(16) = 1.905, *p* = 0.037], and marginally greater *left anodal DLPFC* stimulation (*M* = 0.42, *SD* = 0.07) compared to sham [*M* = 0.39, *SD* = 0.07l; *t*(16) = 1.596, *p* = 0.065].

**TABLE 5:** Averages for memory and metamemory measures by task and stimulation.

		Memory and metamemory test					
**Task**	**Stimulation condition**	**Recall**	**Recognition**	**Average FOK ratings**	**FOK—correct recognition**	**FOK—incorrect recognition**	**d_a_**
Semantic	Left anodal DLPFC	0.27 (0.13)	0.54 (0.14)	5.43 (1.65)	6.12 (1.58)	4.47 (1.73)	−0.1116 (1.03)
	Right anodal DLPFC	0.28 (0.13)	0.56 (0.09)	5.49 (1.59)	6.25 (1.46)	4.43 (1.80)	−0.0947 (0.44)
	Sham	0.29 (0.14)	0.56 (0.11)	5.35 (1.49)	6.13 (1.38)	4.26 (1.59)	−0.1188 (0.56)
Episodic	Left anodal DLPFC	0.04 (0.06)	0.51 (0.13)	4.27 (1.83)	4.63 (1.98)	3.81 (1.76)	0.0202 (0.92)
	Right anodal DLPFC	0.04 (0.05)	0.52 (0.15)	4.11 (1.75)	4.39 (1.85)	3.70 (1.67)	−0.0668 (1.02)
	Sham	0.05 (0.08)	0.52 (0.14)	4.26 (1.94)	4.52 (1.99)	3.90 (1.99)	0.1152 (0.95)

Standard deviations in parentheses.

**FIGURE 4:**
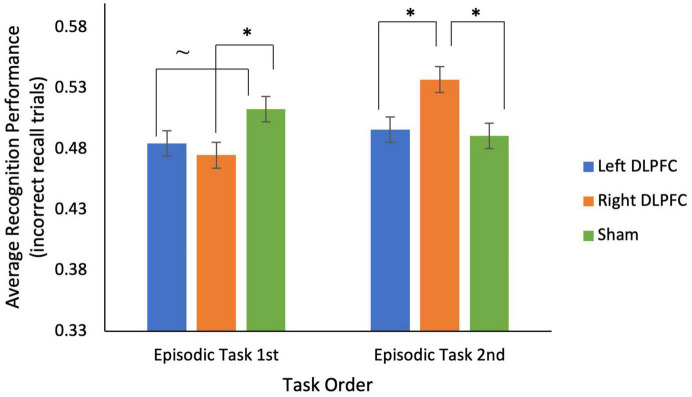
There was a significant interaction between stimulation condition and task order for recognition performance on incorrectly recalled trials for the episodic task. For participants who completed the episodic task 1st, during sessions 1–3, recognition performance was significantly lower for *right* *anodal DLPFC* stimulation compared to sham, and marginally lower for *left* *anodal DLPFC* stimulation compared to sham. For participants who completed the episodic task 1st, during sessions 4–6, recognition performance was significantly higher for *right* *anodal DLPFC* stimulation compared to both *left* *anodal DLPFC* and sham. *p < 0.05, ∼ indicates marginal significance (*p* = 0.08).

For the episodic task, a 2 (episodic task 1st vs. 2nd) × 3 (*Left anodal DLPFC* vs. *Right anodal DLPFC* vs. sham) mixed ANOVA on average recognition performance (for incorrectly recalled trials) showed a significant interaction between stimulation condition and task order [*F*_(2, 68)_ = 4.314, *p* = 0.017; see Figure 4] and no main effects of stimulation condition or task order (all *p*’s > 0.50). This interaction was driven by participants who received the episodic task 1st having significantly greater recognition performance for sham (*M* = 0.51, *SD* = 0.16) compared to *right anodal DLPFC* (*M* = 0.48, *SD* = 0.14) stimulation [*t*(17) = −2.100, *p* = 0.025], and marginally greater recognition performance for sham (*M* = 0.51, *SD* = 0.16) compared to *left anodal DLPFC* (*M* = 0.49, *SD* = 0.15) stimulation [*t*(17) = −1.472, *p* = 0.08]. In contrast, participants who received the episodic task 2nd had significantly greater recognition performance for *right anodal DLPFC* (*M* = 0.54, *SD* = 0.13) compared to *left DLPFC* (*M* = 0.50, *SD* = 0.10) stimulation [*t*(17) = −1.824, *p* = 0.043], and significantly greater recognition performance for *right anodal DLPFC* stimulation (*M* = 0.54, *SD* = 0.13) compared to sham [*M* = 0.49, *SD* = 0.11; *t*(17) = 2.056, *p* = 0.028].

### The effects of rs-tDCS on metamemory

A 2 (semantic task 1st vs. 2nd) × 3 (*Left anodal DLPFC* vs. *Right anodal DLPFC* vs. sham) × 2 (correct vs. incorrect recognition) mixed ANOVA on average FOK ratings for incorrectly recalled trials was conducted to determine how stimulation affected metamemory ratings, and if this differed by task order and recognition accuracy. As expected, there was only a main effect of recognition accuracy [*F*_(1, 33)_ = 124.670, *p* < 0.001], with greater average FOK ratings for correct (*M* = 6.16, *SD* = 1.35) compared to incorrect recognition (*M* = 4.38, *SD* = 1.60).

An analogous test was conducted for the episodic task and showed a main effect of recognition accuracy [*F*_(1, 34)_ = 46.836, *p* < 0.001], with greater average FOK ratings for correct (*M* = 4.51, *SD* = 1.72) compared to incorrect recognition (*M* = 3.80, *SD* = 1.66). A marginal interaction between recognition accuracy and visit order [*F*_(1_,_34)_ = 4.094, *p* = 0.051] was probed and showed that this interaction was driven by a greater mean difference in FOK ratings for participants who had the episodic task 2nd (*Mean difference* = 0.923) vs. those who had the episodic task 1st (*Mean difference* = 0.502). This suggests that participants were more diagnostic of memory performance with their FOK ratings when they had the episodic task second. There were no other significant differences, indicating that FOK ratings were not affected by stimulation or task order.

Mixed ANOVAs on d_a_ for each task were conducted to test how stimulation and task order affected metamemory accuracy. There were no significant main effects of stimulation condition, or task order, and no interaction between stimulation condition and task order for either the semantic or episodic task (all *p*’s > 0.30).

## Discussion

A major goal was to test if prior HD-tDCS findings that stimulation over the left DLPFC increases semantic recognition memory (Chua et al., 2017) and metamemory monitoring accuracy (Chua and Ahmed, 2016; Chua et al., 2017) extended to rs-tDCS for semantic and episodic memory and metamemory tasks. Overall, there were no main effects of stimulation on memory and metamemory. An ancillary goal was to examine order effects in multiple experiment designs with tDCS. Indeed, there were significant differences in recognition performance that were dependent on the order of completing the semantic and episodic tasks and stimulation condition.

Regardless of task type (i.e., semantic vs. episodic), results from the second task were consistent with our original predictions. Participants who had the semantic task second, during sessions 4–6, had better recognition during left DLPFC stimulation compared to sham, consistent with previous findings that HD-tDCS over the left DLPFC can improve recognition performance for a semantic task, during sessions 4–6, (Chua et al., 2017). Participants who had the episodic task second had greater recognition performance for right anodal DLPFC stimulation compared to both left anodal DLPFC stimulation and sham, consistent with prior work showing the involvement of the right DLPFC in episodic retrieval (Rossi et al., 2001; Tulving, 2002; Rami et al., 2003; Blumenfeld and Ranganath, 2007). We can only speculate about why there were differential effects for the first vs. second tasks. One possible explanation relates to comfort with tDCS and practice with the tasks. Participants may have been more comfortable with stimulation and/or the cognitive tasks during their second set of sessions, which could have altered the way tDCS affected their performance. Another possibility relates to the total amount of stimulation received over the course of the study. Some researchers argue that single-session tDCS is not enough to alter cognitive processes, and that more longitudinal, multi-session approaches are warranted (Berryhill, 2017; Talsma et al., 2017; Perceval et al., 2020; Au et al., 2022). In the sessions with the second task, cumulative effects of stimulation could have resulted in the improvements in memory performance seen in these participants. More work comparing single- vs. multi-session tDCS is needed to understand how cumulative effects of tDCS affect different cognitive processes (Inagawa et al., 2019).

Despite the data reflecting our expected pattern of results during the second tasks, the pattern of results shown for participants who completed the semantic or episodic tasks first, during sessions 1–3, was not expected and we can only speculate about why. Perhaps state-dependent factors during the earlier stages of the experiment, such as task motivation and demands of the task (Müller et al., 2022), time of day (Wong et al., 2018), and even circadian rhythms and hormonal cycles (Horvath et al., 2014) influenced performance and the efficacy of tDCS. Though we would expect many of these state-dependent factors to stay the same over the course of the experiment, perhaps as participants became more comfortable with tDCS and participating in the experiment, the effects of these factors were mitigated. In this experiment, time of day is unlikely to have had a large influence because the timing of sessions was kept fairly consistent. Indeed, analyses that included time of day as a covariate did not explain additional variance. Future research on specific state-dependent factors is needed.

Although we showed no effects of anodal rs-tDCS over the DLPFC on metamemory monitoring, it is important to report both the significant and null results shown in this study (Apšvalka et al., 2018; Jacoby and Lavidor, 2018; Westwood and Romani, 2018; Esposito et al., 2022; Reteig et al., 2022). Replication in tDCS studies is often difficult to achieve, as participants often have many individual differences in skull shape/thickness, subcutaneous fat and cerebrospinal fluid densities, as well as anatomical differences in brain regions (Stagg and Nitsche, 2011; Woods et al., 2016). Also, differences in participant arousal (Esposito et al., 2022), time of day (Wong et al., 2018), and even task demands (Chua et al., 2017; Stephens et al., 2017) can influence how participants respond to brain stimulation. By reporting null tDCS results, a clearer picture of which conditions lead to successful tDCS outcome, and which conditions do not, may emerge.

Two ancillary goals were to test the feasibility of fully remotely supervised tDCS without an initial office visit for training, and the feasibility of administering multiple experiments with rs-tDCS. Most rs-tDCS protocols require the first session, or at least the training session, in the office to ensure that participants can properly set up the device (Charvet et al., 2020; Gough et al., 2020; Shaw et al., 2020). Only 1 out of 40 participants failed to complete the remote training session. All other participants were able to successfully implement the protocol, and troubleshoot if needed, with the help of the researcher. Thus, fully remote tDCS is feasible, and in healthy populations an initial office visit may not be needed. Participants were also able to successfully complete both experiments that were administered throughout the course of the experiment. Though we did show order effects for stimulation, overall, performance was as expected for these tasks. Note that an initial in person training session may be necessary or beneficial for other populations, and future studies should address this issue.

One potential limitation is that there were differences in tingling sensations between stimulation conditions for the semantic task and burning sensations in the episodic task. However, our results did not change when conducting the analyses with these sensations as covariates. Furthermore, model fits using generalized mixed models were better without the sensations as covariates. Another potential limitation of this experiment is that the sample consisted of healthy young adults, and thus the results, including feasibility of remotely supervised training, should only be interpreted considering that population. Those interested in aging should refer to studies that utilize older adult populations. Another limitation is that we did not assess performance at baseline, which is used in pretest-posttest designs. Thus, it is possible that comfort or learning about the task contributed to our results. However, because this experiment was designed to examine single session, online effects of tDCS, we used a sham control. Future work with should investigate the effects of multi-session rs-tDCS in a pretest-posttest design.

## Conclusion

Despite not showing an effect of anodal rs-tDCS over the DLPFC on metamemory monitoring accuracy, there was an effect of anodal rs-tDCS on memory performance that varied by task order. Future research should test the effect of multi-session tDCS on cognitive processes to determine how cumulative effects of tDCS impact performance. There is also a need for more studies examining how tDCS effects performance in multiple tasks, as this is more analogous to real life. Finally, more research on the influence of state-dependent factors on tDCS efficacy is needed.

## Data availability statement

The raw data supporting the conclusions of this article will be made available by the authors, without undue reservation.

## Ethics statement

The studies involving humans were approved by the Human Research Protection Program at the City University of New York. The studies were conducted in accordance with the local legislation and institutional requirements. The participants provided their written informed consent to participate in this study.

## Author contributions

CI and EC designed and conducted the study, including the recruitment of participants, the collection and analysis of data, drafted the manuscript was prepared with intellectual input, and complete access to the study data. Both authors approved the final manuscript.

## Footnotes

1 http://www.mangelslab.org/bknorms (Development of the B-KNorms was overseen by Jennifer Mangels and supported by the Network Science Collaborative Technology Alliance of the Army Research Laboratory. Please contact Jennifer Mangels at jennifer.mangels@baruch.cuny.edu for more information concerning the B-KNorms).
